# Shifting Up Cutoff Value of D-Dimer in the Evaluation of Pulmonary Embolism: A Viable Option? Possible Risks and Benefits

**DOI:** 10.1155/2012/517375

**Published:** 2012-07-24

**Authors:** Bennidor Raviv, Shlomo H. Israelit

**Affiliations:** Department of Emergency Medicine, Rambam Health Care Campus, Haifa 31096, Israel

## Abstract

*Objectives.* To evaluate the viability of the possibility to use a higher D-dimer value than the one used today in the clinical algorithms evaluating patients suspected to have pulmonary embolism. *Methods.* A retrospective analysis of 300 serial patients for whom D-dimer values were taken during a 10 month period in the emergency room of a tertiary medical center. *Results.* Our analysis showed that it may be safe and cost effective to use a D-dimer value of 900 ng/ml rather than the value of 500 ng/ml accepted today, with sensitivity of 94.4%. In younger patients [under 40 years] the sensitivity reached was even higher—100%. *Conclusions.* Raising cutoff values of D-dimer in screening for pulmonary embolism seems a viable option. There may be a place for “tailoring” cutoff values according individual patient characteristics, such as according age groups. More studies of the subject are warranted.

## 1. Introduction

Pulmonary embolism is one of the major diagnoses encountered by the emergency physicians.

Pulmonary embolism may present as a wide range of symptoms and signs [1–3]—classically dyspnea and/or chest pain without any other clear cause during evaluation, but also palpitations and unexplained tachycardia, syncope, fever and others—this is the reason why pulmonary embolism is also known as the “great masquerader” [3].

There is no specific clinical sign or ancillary laboratory test for diagnosing pulmonary embolism, and confirmation or ruling out of this condition is based on a combination of clinical suspicion and algorithms that guide whether to proceed the evaluation in a given patient directly with imaging studies if high pretest probability exists based on a clinical scale, or with a blood examination, D-dimer, and then proceeding to imaging studies only if the D-dimer is above a standard cutoff of 500 ng/mL in patients with a low-moderate pretest probability [3, 4].

D-dimer is a fibrin degradation product by plasmin and it represents an endogenic thrombolytic process [3, 5].

It is a nonspecific test, influenced by many factors (patient's age, background illnesses, any inflammatory state, and pregnancy) [3, 5, 6].

By using the currently accepted D-dimer cutoff, many patients are exposed to unnecessary imaging studies, along with their adverse effects, the evaluation time lengthens, and the medical system has to bare extra costs and prolonged waiting times in emergency rooms.

## 2. Aims

By statistically analyzing the results of imaging studies undertaken and the number of positive cases for the diagnosis we tried to find a higher cutoff value of D-dimer than the one used today, while conserving the high sensitivity of the current value on one hand, and reducing unnecessary imaging studies on the other.

As secondary targets we examined the other findings found on imaging studies besides pulmonary embolism for each of the groups—the findings that promoted the diagnosis and treatment of the patient, and those that can be considered as “incidental” findings, that most probably are not related to the patient's clinic but will need further evaluation and followup, may be necessitating further imaging studies and maybe even invasive procedures.

We also looked for differences in D-dimer values across different age groups and genders.

## 3. Methods and Patients

We conducted a retrospective cohort study by analyzing data regarding all the patients for whom D-dimer test was taken in the emergency department of our hospital between 01/01/2010 and 30/10/2010.

Rambam Medical center is a tertiary medical center for the whole of northern Israel.

There are about 70000 admissions to the ER, annually.

Only patients suitable for testing of D-dimer were included: those with suspected clinical presentation—otherwise unexplained dyspnea and/or chest pain, and with low-intermediate pretest clinical probability according to the modified Wells criteria, validated and presented in the work by Wells et al. [4] for predicting the pretest clinical probability of a patient for pulmonary embolism.

D-dimer levels were obtained using the LIA-test D-Di (Stago Diagnostica, Asniéres-sur-Seine, France), the validation of this test was determined elsewhere [7].

For every patient, data was extracted about age, sex, symptoms, and signs for which he or she were undergoing evaluation, D-dimer value, findings in imaging tests if they were eventually undertaken for the patient, including incidental findings and other diagnoses relevant to the patient's acute complaints, and the outcome for the patient in the emergency room setting—hospitalization or discharge.

Statistical analyses were made to determine the sensitivity and specificity of D-dimer in different cutoffs in values between 800 ng/mL and 1000 ng/mL in order to determine the most suitable value.

All patients included underwent imaging studies to diagnose or to rule out pulmonary embolism—the default choice being CT angio.

Lung scan was performed in patients unable to undergo CT (due to allergy to iodine, renal failure, or active asthma in background).

The study was approved by the institutional review board.

## 4. Exclusion Critirea

1. Patients with high probability using the Wells score were drawn out of the study, as they are not candidates for D-dimer testing according the guidelines to begin with.
2. Patients for whom evaluation was incomplete or any required data was insufficient.

## 5. Statistics

Data was analyzed with SPSS version 18 (Statistics Solutions Services).

*T*-Test analysis was performed for the differences between quantitative variables (age and Ddimer,) comparing between categorical variables (sex, CT results, and hospitalization).

Fisher exact test and chi square tests were used for differences between categorical variables.

The Receiver Operating Characteristic (ROC) curve was constructed to describe the relationship between the sensitivity and the false positive rate for different values of Ddimer in the identification of patients suspected to have pulmonary embolism.

Diagnostic parameters (sensitivity, specificity) were used for the relation between D-dimer values and pulmonary embolism diagnosis.

*P* < 0.05 was considered as significant.

## 6. Results

A total of 300 patients with suitable D-dimer values were included.

188 (62.66%) were females and 112 (37.34%) were males.

There was no significant difference in the mean age between females (54.38 ± 19.16) and males (53.57 ± 17.60), *P* = 0.7.

There was no difference in D-dimer values between genders [*P* = 0.53, Table 1].

When the patients were divided into age groups: 65 years old and older, 40–65 years old, and younger than 40, there was a linear relation between the patient's age and D-dimer values [*r* = 0.31, *P* < 0.001], and a statistical difference between the elder patients group and each of the other groups [*P* < 0.001, Table 2].

There is a statistical difference in D-dimer values and age in the three groups, between the eldest patients [over 65 years old], and each of the other groups. *P* < 0.0001 respectfully.

18 of the patients were eventually found to have pulmonary embolism.

In 17 patients other reasons for their symptoms were found on imaging studies, mostly pulmonary congestion or infections.

There was a statistically significant difference between D-dimer values of the patients found to have pulmonary embolism to both the patients with normal imaging studies and those in whom alternative diagnosis was found on imaging studies, with the median D-dimer value higher in the pulmonary embolism group [*P* < 0.001, Table 3].

There is a statistical (nonparametric) difference between groups:

1. alternative diagnoses versus Pulmonary embolism—*P* < 0.0001;
2. no findings versus pulmonary embolism—*P* < 0.0001.

All of the patients diagnosed with pulmonary embolism were hospitalized, as well as 23.5% of the patients with alternative diagnoses found on imaging studies, and 28% of those with no relevant findings on imaging studies.

Unsurprisingly maybe, the D-dimer values for the patients who were eventually discharged from the hospital were significantly lower than for those hospitalized [*P* < 0.001, Table 4].

The patients who were eventually hospitalized had higher D-dimer values than those discharged (2131 ng/mL versus 1260 respectfully, *P* < 0.001).

In order to determine the most appropriate D-dimer value for conserving maximum sensitivity of the test, while trying to somehow improve specificity, we conducted studies for D-dimer values between 800 ng/mL and 1000 ng/mL.

The most appropriate alternative cutoff value found, according these analyses was found to be 900 ng/mL, with sensitivity of 94.4%, and specificity of 49.1% [Table 5].

**Figure 1:**
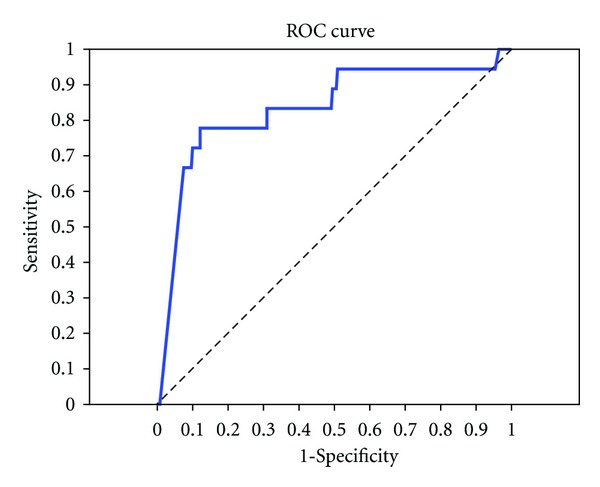
An ROC curve depicting the data. The area under the curve equal 0.835 with 95% Confidence interval 0.72–0.949.

The challenge is to select a cutoff that properly balances the needs of sensitivity and specificity. Thus, approximately 94% of all PE-positive samples would be correctly identified as such, and 51% of all PE-negative samples would be incorrectly identified as positive (Figure 1).

The sensitivity for younger patients (under 40 years old) was higher—100%, with 54.9% specificity [Table 6].

There was no statistically meaningful difference for sensitivity of the cutoff proposed between genders.

There was no significant difference in the mean Wells score between the groups above and below the value of 900 ng/mL.

As with the diagnosis of pulmonary embolism, There was also a significant difference in the alternative diagnoses found for patients in the two groups, in favor of the group with D-dimer above 900 ng/mL, with the main diagnoses being pneumonia and pulmonary congestion [odds 2.05 times higher in the group above the cutoff value, *P* = 0.018].

There were also more incidental findings in the group with D-dimer values above 900 ng/mL [odds of incidental finding 3.64 times higher in the group with D-dimer values above the cut off, *P* = 0.008].

## 7. Discussion

One of the major differential diagnoses of chest pain and dyspnea is pulmonary embolism, the diagnosis or exclusion of which rest upon the combination of clinical suspicion and algorithms based on clinical preprobability for the event. [1, 3–5, 8–11].

In recent years there is a sharp increase in the diagnosis of pulmonary embolism, due to higher awareness of medical staff to the diagnosis, implication of diagnostic algorithms for patients evaluation, combining clinical risk stratification for each patient, laboratory studies [the D-dimer] and imaging studies, and due to high availability of CT scanners [1, 11–13].

As a side effect of this process of higher suspicion and diagnosis of pulmonary embolism, there is also a sharp increase in imaging studies, with some hazardous consequences:

- the connection between exposure to radiation during imaging studies and later malignancies, with up to 3 in 1000 women undergoing chest CT angio in the age of 20 may develop directly related neoplasm 20 years later [14–17];
- unnecessary cases of contrast media nephropathy and allergic reactions to iodine [18];
- economic price for every unnecessary imaging study made [19–21];
- longer waiting times in already overcrowded emergency rooms [13, 21];
- incidental findings on imaging studies necessitating further workup, including even invasive diagnostic procedures [22–24];
- many of the thromboses diagnosed are small and peripheral (subsegmental), and there are still many debates about the clinical significance of such thromboses, the need to treat them versus the complications of such treatment—mostly major bleeding [25, 26].

One of the major reasons for the rates of use of imaging for the evaluation of pulmonary embolism is due to the universal use of D-dimer in current algorithms [6, 13, 27].

D-dimer is a degradation product of fibrinogen by plasmin and is a marker for endogenous, ineffective, fibrinolytic process.

Today, highly sensitive kits for D-dimer are used, but the test is very unspecific, and that is the major drawback of the test [27, 28].

There are already reports about the different sensitivity of D-dimer in different age groups and different risk factors, and it seems that base line values of D-dimer are higher in the elderly [6, 15, 21, 28].

It was already shown also, that using D-dimer increases the testing for, while not increasing the diagnosis of, pulmonary embolism [27]. 

In this work we tried to find an alternative for currently accepted cut off value of D-dimer, in an attempt to keep it as sensitive as possible in, while raising its specificity, in an attempt to lower unnecessary imaging studies.

Although the sensitivity of the proposed value in our work [900 ng/mL] is lower than of the current value used today [94.4% versus 98-99%], it still preserves high sensitivity, and when taking into account the saving of unnecessary radiation exposure in many patients, especially younger ones, this small reduction of sensitivity compared to the current cutoff value is further more demised in its significance.

Still more: when looking in the subpopulation of young patients—those under 40 years old—we see that the sensitivity of the cutoff proposed by us is even higher—100% (and this even before taking into account the already mentioned significance of saving radiation exposure in young patients).

Another possible benefit of CT scanning in the evaluation of possible pulmonary emboli is the ability to diagnose and treat other conditions except pulmonary emboli, but in our work such alternative diagnoses were found in 9 patients with D-dimer cut off values below 900 ng/mL, and most of these were either not life threatening, could be treated on an outpatient basis, or didn't need any specific treatment (i.e., isolated rib fracture), or could be diagnosed otherwise—(COPD by lung function test, pulmonary congestion by revision of chest X-ray)—the proof of this claim is that only 23.5% of these patients were eventually hospitalized—compared to 28% of the patients with no relevant findings found on imaging.

As in previous works, it is seen also in our study that age is a risk factor for thromboembolism.

To conclude, we suggest reevaluating current D-dimer values used in the evaluation for pulmonary embolism in order to make the process more beneficial for medical staff, medical system, and patients.

There is place for “individualizing” D-dimer values for different age groups.

This is yet the largest series of real emergency department patients, of all group ages and of both genders, examining the possibility of changing the most appropriate cutoff value of D-dimer in the evaluation of pulmonary embolism in light of all the experience gathered through more than 20 years since the first presentation of the test for evaluation of pulmonary embolism and application of the Wells criteria.

The limitations of the study are its being retrospective and relatively small in size.

There is need in further studies—larger and prospective—for evaluation of D-dimer cutoff value elevation, maybe individualizing values according to the patient's age.

## Figures and Tables

**Table 1:** Relation between gender and Ddimer values (DD).

Sex	Mean Ddimer value (ng/mL)	*N*	Std. deviation	Median	Minimum	Maximum
F	1510.213	188	1.1654441	.955000	.5100	4.6000
M	1596.071	112	1.1494114	1.020000	.5100	4.7600

Total	1542.267	300	1.1583047	.975000	.5100	4.7600

**Table 2:** Relation between patient's age and Ddimer value.

Age (years)	Mean Ddimer value (ng/mL)	*N*	Std. deviation	Median	Minimum	Maximum
<40	1197.750	80	.8822841	.820000	.5100	4.7600
40–65	1307.121	132	.9681662	.900000	.5100	4.0000
65+	2208.182	88	1.3605877	1.960000	.5200	4.6000

Total	1542.267	300	1.1583047	.975000	.5100	4.7600

There is a linear accordance between age and Ddimer values. *P* < 0.001, *r* = 0.31.

**Table 3:** 

Result	Mean Ddimer value (ng/mL)	*N*	Std. deviation	Median	Minimum	Maximum
No findings	1482.348	264	1.0834436	.980000	.5100	4.7600
Pulmonary embolism	3229.444	18	1.2855783	4.000000	.5400	4.0000
Alternative diagnoses	717.059	17	.1381469	.720000	.5100	.9700

Total	1544.013	299	1.1598507	.970000	.5100	4.7600

**Table 4:** 

Patient's outcome	Mean Ddimer value (ng/mL)	*N*	Std. deviation	Median	Minimum	Maximum
Discharge	1260.493	203	.9971135	.840000	.5100	4760.0
Hospitalization	2131.959	97	1.2519806	1930.000	.5400	4000.0

Total	1542.267	300	1.1583047	.975000	.5100	4760.0

**Table 5:** 

		Results		Total
		Other results	Pulmonary embolism	
	Ddimer values	138	**1**	139
1.00	Under 900 ng/mL	99.2%	.8%	100.0%
	% within result 2	**49.1%**	5.6%	43.6%

	Ddimer values	144	17	161
2.00	Above 900 ng/mL	89.3%	10.7%	100.0%
	% within result 2	53.8%	**94.4%**	56.4%

Count % within	282	18	300
Total	94%	6%	100.0%
	% within result 2	100.0%	100.0%	100.0%

**Table 6:** 

		Imaging findings		Total
		Other results	Pulmonary embolism	
	Ddimer values	39	0	39
	Under 900 ng/mL	100.0%	.0%	100.0%
	% within result 2	**54.9%**	.0%	52.7%

	Ddimer values	32	3	35
	Above 900 ng/mL	91.4%	8.6%	100.0%
	% within result 2	45.1%	**100.0%**	47.3%

	Count	71	3	74
Total		95.9%	4.1%	100.0%
	% within result 2	100.0%	100.0%	100.0%
